# Presepsin in Hepatic Pathology: Bridging the Gap in Early Sepsis Detection

**DOI:** 10.3390/diagnostics15151871

**Published:** 2025-07-25

**Authors:** Dana-Maria Bilous, Mihai Ciocîrlan, Cătălina Vlăduț, Carmen-Georgeta Fierbințeanu-Braticevici

**Affiliations:** 1 Gastroenterology Department, “Carol Davila” University of Medicine and Pharmacy, 050474 Bucharest, Romania; dana-maria.bilous@drd.umfcd.ro (D.-M.B.); catalina.vladut@umfcd.ro (C.V.); carmen.fierbinteanu@umfcd.ro (C.-G.F.-B.); 2 Gastroenterology Clinic, “Prof. Dr. Agrippa Ionescu” Emergency Hospital, 011356 Bucharest, Romania; 3 Internal Medicine II and Gastroenterology Clinic, Emergency University Hospital Bucharest, 050098 Bucharest, Romania

**Keywords:** presepsin, sepsis, liver cirrhosis

## Abstract

Sepsis represents a major cause of mortality, especially among patients with liver cirrhosis, who are at increased risk due to immune dysfunction, gut-derived bacterial translocation, and altered hepatic metabolism. Traditional biomarkers such as C-reactive protein (CRP), procalcitonin (PCT), and interleukin-6 (IL-6) often have reduced diagnostic reliability in this subgroup, due to impaired liver and renal function. Presepsin, a soluble fragment of CD14 released during phagocytic activation, has emerged as a promising biomarker for early sepsis detection. This systematic review explores the diagnostic and prognostic utility of presepsin in cirrhotic and non-cirrhotic patients with suspected infection. Data from multiple clinical studies indicate that presepsin levels correlate with infection severity and clinical scores such as SOFA and APACHE II. In cirrhotic patients, presepsin demonstrates superior sensitivity and specificity compared to conventional biomarkers, maintaining diagnostic value despite hepatic dysfunction. Its utility extends to differentiating bacterial infections from fungal infections and monitoring treatment response. While preliminary evidence is compelling, further prospective, multicenter studies are required to validate its integration into standard care algorithms. Presepsin may become a valuable addition to clinical decision-making tools, particularly in hepatology-focused sepsis management.

## 1. Introduction

Sepsis is a critical clinical condition defined by the Third International Consensus Definitions for Sepsis and Septic Shock (Sepsis-3) as a “life-threatening organ dysfunction caused by a dysregulated host response to infection” [1]. Physiologically, sepsis involves the early activation of both pro-inflammatory and anti-inflammatory responses, alongside interactions with various systems such as coagulation and endocrine pathways. These responses are initiated by the innate immune system in response to a pathogen [1,2,3]. The severity of sepsis can be assessed using the Sequential Organ Failure Assessment (SOFA) score, where an increase of two points or more correlates with a 10% rise in 28-day mortality rates [4].

Liver cirrhosis is a significant risk factor for infections, with its complications contributing to a 63% annual mortality rate [5]. Bacterial infections are a primary trigger for early acute liver decompensation, occurring 4–5 times more frequently than other organ decompensations. Moreover, patients with cirrhosis face a twofold higher risk of developing sepsis compared to those with other diseases [5,6]. This elevated risk arises from the complex pathophysiology of cirrhosis, which extends beyond liver damage. Recent studies emphasize the pivotal role of gut health and microbiota. Research by Maslennikov et al. highlights that gut dysbiosis and small intestinal bacterial overgrowth (SIBO) contribute directly to a systemic pro-inflammatory state, leading to an imbalance in cytokine regulation, often termed a “cytokine storm” [7]. Cytokine storms exacerbate immune dysregulation in cirrhotic patients, making them more prone to severe infections. Consequently, cirrhotic patients exhibit an exaggerated immune response to infections, typically progressing through the stages of sepsis, severe sepsis, and septic shock [8,9].

This phenomenon is further aggravated by impaired intestinal barrier function and altered immune cell surveillance in the mesenteric lymphatic system. Such alterations create a vicious cycle of endotoxemia and immune overactivation.

Moreover, the systemic inflammatory response in cirrhosis often masks the clinical presentation of infections, delaying diagnosis and increasing mortality risk. Classical signs such as fever or leukocytosis may be absent due to immune paresis. Therefore, reliance on biomarkers with high sensitivity and specificity becomes critical.

In the past five years, over 200 biomarkers have been identified for their potential role in diagnosing and managing sepsis. Biomarkers are defined as reliable tools that can be objectively measured and evaluated to indicate normal or pathological processes. An ideal biomarker should be disease-specific and highly sensitive, offering prognostic insights, reflecting clinical progression and therapeutic responses, and being cost-effective and easy to measure [10,11].

Various biomarkers have been investigated over the past decade to identify the most effective ones for sepsis in cirrhotic patients. These include white blood cell count (WBC), C-reactive protein (CRP), procalcitonin (PCT), and presepsin (PSP), among others [2]. It is worth noting that WBC levels are linked to the degree of hypersplenism in cirrhotic patients. CRP, an acute-phase protein produced by the liver, depends on liver function, while PCT, a precursor of calcitonin, is influenced by renal clearance, making its serum levels susceptible to renal impairment [5].

Before discussing the role of presepsin as a biomarker for sepsis in cirrhotic patients, it is important to understand its formation. When pathogens enter the human body, they express specific motifs known as microorganism-associated molecular patterns (MAMPs).

Cluster of Differentiation 14 (CD14), a member of the Toll-like Receptor (TLR) family found on monocytes and macrophages, exists in two forms: a membrane-bound form (mCD14) and a soluble form (sCD14). Acting as a co-receptor, CD14 identifies pathogens by recognizing their specific MAMPs, also referred to as ligands. The most well-studied ligand for CD14 is lipopolysaccharide (LPS), a component of the outer membrane of Gram-negative bacteria. Once LPS binds to CD14, it is presented to TLRs, which then activates immune cells to initiate an immune response.

During this process, proteases and cathepsin D cleave and release the soluble form of CD14 (sCD14) into the bloodstream. The N-terminal fragment of sCD14, known as presepsin, has been of particular interest since its discovery in 2004, especially for its potential role in the early diagnosis of sepsis [12].

Over the past decade, significant efforts have been directed toward refining diagnostic tools, and, among the multitude of biomarkers evaluated, presepsin has emerged as a particularly promising candidate, offering both pathophysiological relevance and clinical applicability.

## 2. Materials and Methods

### 2.1. Search Strategy and Protocol

**Figure 1:**
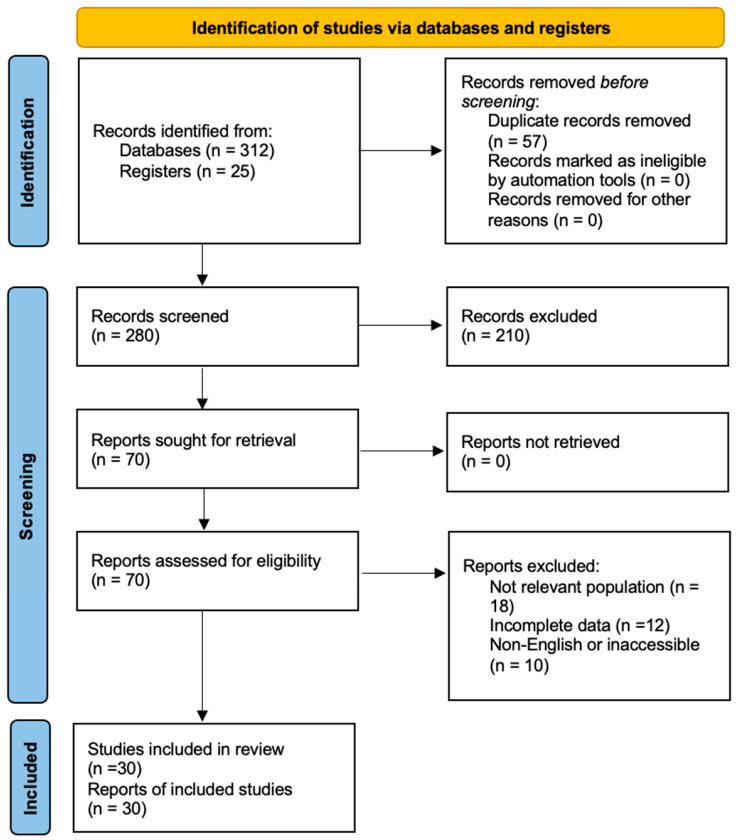
PRISMA flow diagram of the study selection process.

A systematic review and meta-analysis of the literature was conducted following the PRISMA (Preferred Reporting Items for Systematic Reviews and Meta-Analysis) guideline from 2020 [13]. Many literature searches were performed during January 2025 and March 2025 in the PubMed, Google Scholar, and Elsevier Science Direct databases using the following search terms: presepsin, sepsis, cirrhosis, and liver failure. We searched for reviews and clinical studies performed on humans in the last fifteen years that evaluated biomarkers in septic, infected, or non-infected cirrhotic patients for their use in diagnosis and therapeutic management. We included peer-reviewed full-text articles written in English (Figure 1). The applicable articles were reviewed to evaluate any supplementary studies. Their references might have been overlooked during the systematic research.

The first literature search was conducted on 7 January 2025 by two reviewers, which consisted of reviewing titles and abstracts for their eligibility. Editorials, book sections, duplicated articles, conference reports, and case reports were excluded. The risk of bias was assessed for each included study using predefined methodological criteria adapted from established guidelines, focusing on aspects such as study design, sample size, blinding, and potential conflicts of interest. Three reviewers independently evaluated the studies, and discrepancies were resolved through discussion and consensus. No automation tools were used in this process.

### 2.2. Data Extraction

The following data were extracted by four independent investigators (D.B., C.V., M.C., C.F.): year, design of study, review type, variations in presepsin values correlated with the infected/non-infected status, the stage of sepsis (sepsis, severe sepsis, or septic shock), the stage of liver damage (healthy liver, presence of liver illness, and cirrhotic stage—compensated or decompensated), and any other important findings of the studies and reviews.

A narrative assessment of individual study quality was also performed. Most included studies were observational, with moderate to high risk of bias due to the lack of blinding, small sample sizes, and absence of randomization. Three studies were rated as low risk based on robust methodology and complete reporting.

Ultimately, a total of 28 studies met the inclusion criteria and were analyzed in this review.

Any disagreement encountered was discussed until final consensus.

### 2.3. Objective

The primary aim of this review was to evaluate the diagnostic utility of presepsin as an early biomarker in septic cirrhotic patients. A secondary objective was to explore the variations in presepsin levels among infected and non-infected patients with liver conditions, both with and without cirrhosis.

### 2.4. Definitions

In this review, presepsin (PSP) refers to the N-terminal 13 kDa fragment of the soluble subtype of CD14, which appears in human serum following immune activation triggered by pathogens. Sepsis is a term used to describe the body’s severe immune response to infection and is classified into three stages: sepsis, severe sepsis, and septic shock. Cirrhosis, on the other hand, represents the advanced stage of chronic liver damage, often resulting from viral infections or toxic exposure.

### 2.5. Bias Considerations

To minimize reporting bias, we evaluated the potential for selective publication or outcome reporting by comparing study protocols, where available, with published data. Funnel plot asymmetry and the presence of missing outcome data were considered in narrative synthesis. However, due to the qualitative nature of the synthesis and limited availability of unpublished protocols, formal statistical assessment of reporting bias was not feasible.

The overall certainty of the evidence was not formally assessed using a structured tool such as GRADE. However, study quality, consistency of findings, and indirectness were considered during interpretation. Limitations such as small sample sizes and heterogeneity among included studies reduce the overall confidence in the conclusions.

## 3. Results

- Presepsin in Sepsis

Sepsis is a high-incidence condition with elevated mortality rates, particularly among critically ill patients. Prompt and accurate diagnosis is essential for the initiation of appropriate treatment. Presepsin (sCD14-ST) has recently emerged as a promising biomarker for the early diagnosis of sepsis, outperforming procalcitonin (PCT) and C-reactive protein (CRP) in some studies. Presepsin is a soluble fragment of CD14 released during the phagocytosis of microorganisms. It is released from monocytes and macrophages upon phagocytosis of pathogens and appears in circulation within 1–2 h following an infectious stimulus, peaking at approximately 3 h. This rapid response precedes that of traditional biomarkers such as procalcitonin (PCT) or C-reactive protein (CRP), providing an opportunity for earlier detection of septic episodes. Compared to presepsin, procalcitonin (PCT) typically peaks at 6–12 h, and C-reactive protein (CRP) peaks between 24 and 48 h. Furthermore, presepsin exhibits a short half-life of approximately 5 h and a rapid decline following appropriate therapy, allowing for real-time monitoring of disease progression and therapeutic response [14,15].

Clinical studies have demonstrated the high diagnostic accuracy of presepsin. In emergency department settings, presepsin showed comparable or superior diagnostic performance relative to PCT (AUC for presepsin: 0.946 vs. PCT: 0.905), particularly when used in combination with early warning scores (EWS). Additionally, presepsin levels correlate with the severity of infection, exhibiting significantly higher concentrations in patients with septic shock than in those with sepsis alone [14]. Based on the current literature, presepsin cutoff values have been proposed to aid in the clinical interpretation of the results, as follows: <300 pg/mL: sepsis unlikely; 300–500 pg/mL: infection suspected, observation recommended; and >500 pg/mL: high risk, immediate treatment advised. These cutoffs levels may support clinicians in stratifying infection risk and deciding timely therapeutic interventions, especially in complex patients with underlying liver disease [14,16]. According to the study conducted by Ponte et al., the mean presepsin levels in patients with confirmed sepsis were 2926 pg/mL, compared to 1749 pg/mL in those with non-infectious SIRS. The ROC analysis yielded an AUC of 0.787 (95% CI: 0.686–0.889), supporting good sensitivity and specificity for sepsis diagnosis [17].

Another observational clinical study carried out in an ICU setting identified a significant positive correlation between presepsin levels and both APACHE II (r = 0.54, *p* = 0.0008) and SOFA scores (r = 0.54, *p* = 0.0006) on admission day, suggesting that presepsin may serve as a marker of the clinical severity of sepsis [16,17].

Furthermore, according to a systematic review by Struyf et al., although procalcitonin was the most frequently studied marker, its sensitivity in emergency settings among elderly patients was limited, and combining multiple markers did not significantly improve the diagnostic performance. This underscores the need for more sensitive biomarkers such as presepsin [18].

The short turnaround time for obtaining results renders presepsin a valuable tool for rapid diagnosis in emergency and intensive care settings. Kaul and Shah emphasized the clinical need for biomarkers with high specificity and rapid response time characteristics met by presepsin [19].

In addition, its clinical utility is further validated in pediatric and neonatal populations, where symptoms are often non-specific and other diagnostic approaches may lead to overdiagnosis or unnecessary treatments. Importantly, presepsin also appears to hold pan-demographic applicability, showing reliable performance across age groups, including neonates, adults, and the elderly. In neonates, presepsin levels were significantly elevated in septic patients compared to non-septic controls, with cutoff values ranging between 650 and 850 pg/mL, and correlated with survival outcomes. In elderly populations, who exhibit atypical presentations and are particularly vulnerable to sepsis-related mortality, presepsin may enable earlier diagnosis and stratification of risk, even when classical signs are absent [20].

Despite these promising findings, challenges remain regarding the standardization of assay techniques, cost constraints, and the absence of consensus cutoff values, which limit routine implementation in clinical practice. A multi-marker approach incorporating presepsin, PCT, IL-6, and CRP may offer superior diagnostic precision and overcome the limitations inherent in any single biomarker [15,21].

In meta-analytic comparisons, presepsin demonstrated a pooled sensitivity of 75% and specificity of 80% in cirrhotic patients for detecting bacterial infections, closely paralleling the performance of PCT. In critically ill populations, presepsin has also shown value in distinguishing between bacterial and non-bacterial causes of inflammation, with cutoff values around 869 pg/mL for Gram-negative DNAemia detection via metagenomic sequencing techniques [15,22].

Presepsin proves to be a promising biomarker for the early diagnosis of sepsis, with diagnostic accuracy that is comparable or even superior to traditional markers. Its rapid detection time, correlation with disease severity, and prognostic potential make presepsin a valuable clinical instrument.

- Presepsin in Bacterial and Fungal Infections

In a recent narrative review, Noppè et al. discuss the rising incidence of fungal infections in ICU, particularly candidemia, and the challenges of early diagnosis [23]. Although presepsin is not specifically for fungal infections, its combination with other markers, such as 1,3-β-D-glucan (BDG), may enhance diagnostic sensitivity for invasive candidiasis (IC). This combination is especially useful in high-risk settings such as immunosuppression, prolonged antibiotic use, or the presence of central venous catheters.

Presepsin tends to reach higher levels and rises more rapidly in bacterial infections, especially those caused by Gram-negative bacilli, compared to fungal infections. In fungal infections, presepsin elevation is more modest and delayed. Therefore, dynamic monitoring of this biomarker can assist in etiological differentiation.

In contrast to BDG, which has limited specificity but an excellent negative predictive value (NPV > 90%) for candidiasis, presepsin provides an early signal of general septic status. Elevated presepsin levels in a patient colonized with Candida may suggest bacterial superinfection, warranting further etiological investigation and reassessment of antimicrobial therapy [24].

- Presepsin in Hepatology

Advanced liver pathology, particularly cirrhosis and acute-on-chronic liver failure (ACLF), is characterized by an increased vulnerability to bacterial infections, which may trigger or exacerbate multiorgan dysfunction. The rapid diagnosis of such infectious complications is essential to reducing mortality.

Efremova et al. (2024) [25] investigated presepsin in cirrhotic patients with no clinically evident infection. Their study demonstrated elevated presepsin levels in cirrhotic patients compared to controls, correlating with Child–Pugh class, bilirubin levels, and hypoalbuminemia. These findings suggest that presepsin reflects subclinical bacterial translocation via the gut–liver axis—a frequent phenomenon in advanced liver disease. Additionally, this study assessed the response to probiotic treatment (Saccharomyces boulardii). Only patients with elevated presepsin levels showed significant clinical improvement after probiotic administration, implying a potential role for presepsin in identifying patients who may benefit from microbial therapy. This novel approach positions presepsin as a predictive marker for targeted intervention in cirrhosis [25].

Bacterial infections represent the leading trigger for ACLF and are a common complication in its clinical course. According to the synthesis provided by Xu Z. et al., the global prevalence of bacterial infections in ACLF patients ranges from 55% to 81%, with incidence increasing in proportion to disease severity [26]. Nosocomial infections, particularly spontaneous bacterial peritonitis (SBP), pneumonia, and urinary tract infections, are predominant.

A systematic review published in the World Journal of Hepatology highlights that presepsin, alongside resistin, demonstrates diagnostic performance comparable to procalcitonin in detecting bacterial infections among decompensated cirrhosis patients admitted to intensive care units [8]. Presepsin has shown strong diagnostic utility in ICU settings. According to Formenti et al. (2024) [27], presepsin is released rapidly in response to bacterial phagocytosis and offers higher specificity for sepsis than conventional markers. While procalcitonin and CRP rise in response to general systemic inflammation, presepsin specifically reflects monocyte/macrophage immune activation. Numerous studies suggest that presepsin levels correlate with sepsis severity and decrease following effective antibiotic therapy. Although cutoff values vary, recent meta-analyses have consistently shown high sensitivity and specificity for presepsin. However, Formenti et al. also emphasize the need for standardized thresholds and multicenter validation before routine clinical implementation becomes feasible [27].

Sepsis-induced cholestasis represents a form of hepatic dysfunction driven by systemic inflammation and hepatic hypoperfusion. According to Ghenu et al., this condition is mediated through downregulation of hepatocellular transporter proteins such as NTCP, BSEP, and MRP2, which negatively impact biliary excretion of bile acids and bilirubin [28]. These mechanisms support the clinical utility of specific biomarkers like presepsin in recognizing subclinical infections. Jin Xu’s doctoral thesis applied metabolomics to human and murine hepatic tissue, identifying endogenous metabolites relevant to systemic hepatic inflammation [29].

In patients with liver pathology complicated by bacterial infections, multiple biomarkers have been investigated for their diagnostic and prognostic value. Procalcitonin (PCT), a frequently used marker, shows moderate sensitivity and specificity in this context. Studies indicate that, while PCT may guide antibiotic therapy, its levels can be adversely affected by severe hepatic dysfunction, limiting its diagnostic accuracy. By contrast, presepsin demonstrates comparable or superior sensitivity and specificity to PCT, even within the immunocompromised milieu of cirrhosis. Furthermore, in comparison with CRP or IL-6, presepsin exhibits a faster kinetic response, being detectable within the early hours of infection onset, whereas CRP may remain non-specifically elevated. IL-6, although a potent inflammatory marker, is seldom used in clinical practice due to high cost and interindividual variability. This performance suggests that presepsin may be effectively employed not only in infection diagnosis, but also in risk stratification, therapy monitoring, and the optimization of antibiotic initiation timing, in conjunction with scoring systems such as SOFA or MELD. It should be noted that presepsin levels can be falsely elevated in patients with renal dysfunction, such as hepatorenal syndrome (HRS), acute kidney injury (AKI), or chronic kidney disease (CKD), conditions frequently encountered in cirrhotic populations. This cofounding factor warrants cautious interpretation. Presepsin emerges as a valuable biomarker in the diagnosis and prognosis of bacterial infections in patients with chronic or acute liver diseases [8,28]. The available evidence supports its integration into clinical algorithms alongside scoring systems such as SOFA, CLIF-SOFA, and MELD, and complementary markers such as procalcitonin or resistin. Future research should validate these findings in prospective clinical studies and explore its integration into standardized management protocols.

In a prospective case–control study, Ghoneim et al. (2024) [30] evaluated the diagnostic role of presepsin in chronic liver disease (CLD) patients with bacterial sepsis. Presepsin levels were significantly elevated in infected individuals compared to healthy controls. This study found that presepsin outperformed CRP and total leukocyte count (TLC) in diagnostic accuracy. At a cutoff level of 0.89 mg/L, presepsin yielded a sensitivity of 91.67% and specificity of 63.33%, making it a potentially superior early diagnostic tool in CLD patients, where standard inflammatory markers are often unreliable due to immune dysregulation [30].

While presepsin was not directly evaluated, a study by Zhang et al. (2021) [31] developed a predictive model for bacterial infections in hepatitis B virus-related acute-on-chronic liver failure. This model incorporated inflammatory parameters, including CRP, IL-6, and globulin levels. The inclusion of presepsin in such prognostic tools could further enhance diagnostic precision, particularly in patient populations with immune dysfunction, systemic inflammation, and high infection risk [31].

Jang et al. (2024) [32] emphasized the limitations of current sepsis diagnostic frameworks and highlighted the need for biomarkers that can reliably differentiate bacterial sepsis from systemic inflammatory response syndrome (SIRS). Presepsin was identified as one such candidate due to its specificity and rapid kinetics. This review also advocated for incorporating presepsin into rapid diagnostic platforms and point-of-care tools, which could facilitate timely clinical decision making and optimize antimicrobial stewardship [32].

## 4. Discussion

The potential for reporting bias at the synthesis level was considered; however, due to the narrative nature of the review and limited access to unpublished data, we were unable to formally assess publication bias.

The utility of presepsin as a sepsis biomarker is gaining increasing recognition due to its robust diagnostic and prognostic characteristics across diverse clinical populations, including those with underlying liver dysfunction. The rapid increase in presepsin levels—detectable within 2 h of pathogen exposure—confers a critical advantage in early diagnosis, especially when juxtaposed against conventional biomarkers such as CRP and PCT, which often exhibit delayed kinetics and are susceptible to confounding by organ dysfunction, particularly hepatic or renal failure [14,15].

In patients with cirrhosis, the diagnostic landscape for sepsis is markedly complex. Cirrhosis is associated with profound immunological dysregulation, characterized by impaired innate immunity, intestinal bacterial translocation, and systemic inflammation. These mechanisms predispose cirrhotic individuals to infections that are both frequent and clinically silent at onset [20,22]. Traditional inflammatory markers such as CRP and PCT are often unreliable in this context due to impaired hepatic synthesis and altered clearance, respectively. By contrast, presepsin is released primarily by monocytes and macrophages upon phagocytosis and is less dependent on hepatic metabolism or renal function, rendering it particularly suitable for use in liver-impaired populations [15,20].

Moreover, the prognostic significance of presepsin is supported by its correlation with established clinical severity scores such as SOFA and APACHE II. Elevated levels on admission have been associated with higher in-hospital mortality, especially among ICU patients with advanced liver disease [14,15]. Its short half-life (approximately 5 h) and prompt decline with effective therapy allow presepsin to function not only as a diagnostic tool, but also as a real-time indicator of therapeutic response. A decrease of more than 30–50% in presepsin levels within 72 h of initiating antibiotic therapy has been proposed as a potential indicator of effective treatment response. These features position presepsin as a dynamic biomarker capable of informing antimicrobial stewardship decisions and reducing unnecessary broad-spectrum antibiotic use, particularly in settings of diagnostic uncertainty [33].

In hepatology, presepsin has shown promise beyond its infectious diagnostic capabilities. Its baseline elevation in non-infected cirrhotic patients likely reflects subclinical endotoxemia and bacterial translocation, phenomena well-documented in the pathophysiology of advanced liver disease [21]. Notably, patients with elevated presepsin levels may represent a subgroup with heightened inflammatory tone and immune activation, for whom preemptive or adjunctive microbial-modulating therapies (e.g., probiotics, selective intestinal decontamination) may offer benefit. Such insights support a precision medicine approach wherein presepsin could aid in identifying patients who might benefit from early interventions, even in the absence of overt clinical infection [21].

Nevertheless, challenges to the widespread adoption of presepsin remain. Chief among them is the lack of universally accepted cutoff values, which vary significantly depending on assay methods, patient populations, and infection types. Furthermore, while presepsin demonstrates better specificity than CRP and is often comparable or superior to PCT, its performance in fungal infections appears limited unless used in conjunction with other markers such as 1,3-β-D-glucan. Additionally, the cost and availability of presepsin assays may impede integration into resource-limited settings, despite their potential utility. Emerging evidence suggests that combining presepsin with other markers and clinical scoring systems may enhance diagnostic accuracy. Multi-marker strategies that integrate presepsin with IL-6, PCT, and CRP, in tandem with scoring tools such as CLIF-SOFA or MELD, may allow better risk stratification and treatment optimization. Future multicentric studies should validate such algorithms and assess their impact on clinical outcomes [21].

These features position presepsin as a dynamic biomarker capable of informing antimicrobial stewardship decisions and reducing unnecessary broad-spectrum antibiotic use, particularly in settings of diagnostic uncertainty [33]. In addition, the kinetic profile of presepsin supports its use not only at a single time point but also as part of serial monitoring strategies to assess therapeutic efficacy and predict outcomes.

Emerging data suggest that presepsin may also play a role in differentiating bacterial infections from other causes of systemic inflammation, such as viral infections or autoimmune flares, especially in immunocompromised patients. The potential application of presepsin in non-infectious inflammatory liver conditions (e.g., alcoholic hepatitis, autoimmune hepatitis) remains an area worth exploring, given its specificity for microbial-derived immune activation.

Moreover, the integration of presepsin into rapid point-of-care diagnostic platforms is under investigation. These developments could drastically improve turnaround times in emergency departments and ICUs, allowing clinicians to make informed decisions earlier in the disease course. While current data are promising, particularly in Asian and European cohorts, broader validation across geographic, ethnic, and clinical subgroups remains necessary. Another key challenge is the cost-effectiveness of routine presepsin testing, especially in comparison with other biomarkers like CRP and PCT. In addition, in low-resource or remote settings, the availability of point-of-care presepsin assays would be essential for timely decision making.

While presepsin consistently demonstrated high sensitivity and specificity across studies, the certainty of evidence remains moderate due to heterogeneity in study design and variability in diagnostic thresholds. Standardized multicenter trials are needed to increase the confidence in its clinical applicability.

## 5. Conclusions

Presepsin has emerged as a promising biomarker for the early diagnosis and prognosis of sepsis, offering distinct advantages over traditional inflammatory markers such as procalcitonin and CRP. Its rapid kinetics, specificity for monocyte/macrophage activation, and relative independence from hepatic synthetic function make it particularly valuable for patients with chronic liver disease, including those with cirrhosis and acute-on-chronic liver failure. The biomarker demonstrates strong diagnostic performance across diverse populations and clinical settings and may provide additional utility in monitoring therapeutic response and guiding antimicrobial stewardship.

Despite its potential, several barriers to routine clinical implementation remain, including assay cost, variability in cutoff values, and the need for standardization across laboratories. Nonetheless, the current evidence supports the integration of presepsin into multi-parameter diagnostic frameworks alongside clinical scores such as SOFA and MELD, particularly in high-risk, immunocompromised patients. Future large-scale prospective studies are warranted to validate its use in clinical algorithms and to establish clear thresholds for infection diagnosis, risk stratification, and outcome prediction in both general and hepatology-focused critical care settings.

Future large-scale, prospective studies are warranted to validate its use in clinical algorithms and to establish clear thresholds for infection diagnosis, risk stratification, and outcome prediction in both general and hepatology-focused critical care settings. Furthermore, comparative cost-effectiveness analyses between presepsin-based strategies and conventional diagnostic pathways may help to clarify its role in health policy and resource allocation.

Ultimately, the inclusion of presepsin into clinical protocols represents a promising step toward precision medicine in sepsis care, particularly for patients with chronic liver disease who present unique diagnostic challenges. As research continues to evolve, presepsin may prove to be not only a biomarker of infection, but also a surrogate marker for therapeutic response and a predictive tool for tailored interventions.

## Abbreviations

The following abbreviations are used in this manuscript:

SOFA	Sequential Organ Failure Assessment
CRP	C-Reactive Protein
PSP	Presepsin
PCT	Procalcitonin
WBC	White Blood Cell count
MAMPs	Microorganism-associated molecular patterns
CD14	Cluster of Differentiation 14
TLR	Toll-Like Receptor
LPS	Lipopolysaccharide
SIBO	Small Intestinal Bacterial Overgrowth
mCD14	Membrane-bound form CD14
sCD14	Soluble form of CD14
PRISMA	Preferred Reporting Items for Systematic Reviews and Meta-Analysis
ICU	Intensive Care Unit
APACHE	Acute Physiology and Chronic Health Evaluation
ACLF	Acute-on-Chronic Liver Failure
SBP	Spontaneous Bacterial Peritonitis
MELD	End-Stage Liver Disease score
CLIF-SOFA	Chronic Liver Failure–Sequential Organ Failure
BDG	1,3-β-D-glucan
IC	Invasive Candidiasis
NPV	Negative Predictive Value
CLD	Chronic Liver Disease
TLC	Total Leukocyte Count
SIRS	Systemic Inflammatory Response Syndrome
EWS	Early Warning Scores
HRS	Hepatorenal Syndrome
CKD	Chronic Kidney Disease

## References

[1] Singer M., Deutschman C.S., Seymour C.W., Shankar-Hari M., Annane D., Bauer M., Bellomo R., Bernard G.R., Chiche J.-D., Coopersmith C.M. (2016). The third international consensus definitions for sepsis and septic shock (sepsis-3). JAMA.

[2] Jekarl D.W., Lee S., Kim M., Kim Y., Woo S.H., Lee W.J. (2019). Procalcitonin as a prognostic marker for sepsis based on SEPSIS-3. J. Clin. Lab. Anal..

[3] Daniel M., Bedoui Y., Vagner D., Raffray L., Ah-Pine F., Doray B., Gasque P. (2022). Pathophysiology of Sepsis and Genesis of Septic Shock: The Critical Role of Mesenchymal Stem Cells (MSCs). Int. J. Mol. Sci..

[4] Lambden S., Laterre P.F., Levy M.M., Francois B. (2019). The SOFA score—Development, utility and challenges of accurate assessment in clinical trials. Crit. Care.

[5] Igna R., Gîrleanu I., Cojocariu C., Muzîca C., Huiban L., Sfarti C., Cuciureanu T., Chiriac S., Sîngeap A.-M., Petrea O.C. (2022). The Role of Presepsin in Diagnosing Infections in Patients with Liver Cirrhosis and Overt Hepatic Encephalopathy. Diagnostics.

[6] Mitra M., Mancuso A., Politi F., Maringhini A. (2020). Bacterial infections in cirrhosis: A narrative review and key points for clinical practice. Ital. J. Med..

[7] Efremova I., Maslennikov R., Medvedev O., Kudryavtseva A., Avdeeva A., Krasnov G., Romanikhin F., Diatroptov M., Fedorova M., Poluektova E. (2024). Gut Microbiota and Biomarkers of Intestinal Barrier Damage in Cirrhosis. Microorganisms.

[8] Ndomba N., Soldera J. (2023). Management of sepsis in a cirrhotic patient admitted to the intensive care unit: A systematic literature review. World J. Hepatol..

[9] Gustot T., Durand F., Lebrec D., Vincent J., Moreau R. (2009). Severe sepsis in cirrhosis. Hepatology.

[10] Saxena J., Das S., Kumar A., Sharma A., Sharma L., Kaushik S., Srivastava V.K., Siddiqui A.J., Jyoti A. (2024). Biomarkers in sepsis. Clin. Chim. Acta.

[11] Biomarkers Definitions Working Group (2001). Biomarkers and surrogate endpoints: Preferred definitions and conceptual framework. Clin. Pharmacol. Ther..

[12] Azim A. (2021). Presepsin: A promising biomarker for sepsis. Indian J. Crit. Care Med..

[13] Page M.J., McKenzie J.E., Bossuyt P.M., Boutron I., Hoffmann T.C., Mulrow C.D., Shamseer L., Tetzlaff J.M., Akl E.A., Brennan S.E. (2021). The PRISMA 2020 statement: An updated guideline for reporting systematic reviews. BMJ.

[14] Piccioni A., Baroni S., Rozzi G., Belvederi F., Leggeri S., Spagnuolo F., Novelli M., Pignataro G., Candelli M., Covino M. (2025). Evaluation of Presepsin for Early Diagnosis of Sepsis in the Emergency Department. J. Clin. Med..

[15] Zhou Y., Ren D., Chen Y., Wen S., Zhang Y., Song F., Yang M., Eisenhut M., O’rourke J., Li Y. (2025). Presepsin, procalcitonin, interleukin-6, and high-sensitivity C-reactive protein for predicting bacterial DNAaemia among patients with sepsis. J. Thorac. Dis..

[16] Ponte S.T.D., Andrioli G., Diogo L.P., Seligman R., Goldani L.Z., Weber A.P., Rezende G.P., Machado P.S. (2017). Diagnostic Value of Presepsin in Sepsis. Biomark. Appl..

[17] Sharma S.K., Rohatgi A., Bajaj M., Sprung C.L., Morales R.C., Kasdan H., Reiter A., Volker T., Meissonnier J., Beloborodova N. (2016). Sepsis 2016 Agra, India. Crit. Care.

[18] Struyf T., A Boon H., van de Pol A.C., Tournoy J., Schuermans A., Verheij T.J.M., Verbakel J.Y., Bruel A.V.D. (2021). Diagnosing serious infections in older adults presenting to ambulatory care: A systematic review. Age Ageing.

[19] Shah S., Kaul A. (2018). Biomarkers in sepsis. J. Pediatr. Crit. Care.

[20] de Moura E.L.B., Pereira R.W. (2024). Crossing Age Boundaries: The Unifying Potential of Presepsin in Sepsis Diagnosis Across Diverse Age Groups. J. Clin. Med..

[21] Sinha A., Sankanagoudar S., Sharma A., Kothari N., Gupta N., Sharma P. (2025). Recent Scenario of Diagnostic and Prognostic Biomarkers of Sepsis in Clinical Practice and the Role of Multi-marker Approach: An Update. Ann. Afr. Med..

[22] Wejnaruemarn S., Susantitaphong P., Komolmit P., Treeprasertsuk S., Thanapirom K. (2025). Procalcitonin and presepsin for detecting bacterial infection and spontaneous bacterial peritonitis in cirrhosis: A systematic review and meta-analysis. World J. Gastroenterol..

[23] França A. (2023). The Role of Coagulase-Negative Staphylococci Biofilms on Late-Onset Sepsis: Current Challenges and Emerging Diagnostics and Therapies. Antibiotics.

[24] Noppè E., Eloff J.R.P., Keane S., Martin-Loeches I. (2024). A Narrative Review of Invasive Candidiasis in the Intensive Care Unit. Ther. Adv. Pulm. Crit. Care Med..

[25] Efremova I., Maslennikov R., Poluektova E., Medvedev O., Kudryavtseva A., Krasnov G., Fedorova M., Romanikhin F., Zharkova M., Zolnikova O. (2024). Presepsin as a Biomarker of Bacterial Translocation and an Indicator for the Prescription of Probiotics in Cirrhosis. World J. Hepatol..

[26] Xu Z., Zhang X., Chen J., Shi Y., Ji S. (2024). Bacterial Infections in Acute-on-chronic Liver Failure: Epidemiology, Diagnosis, Pathogenesis, and Management. J. Clin. Transl. Hepatol..

[27] Formenti P., Gotti M., Palmieri F., Pastori S., Roccaforte V., Menozzi A., Galimberti A., Umbrello M., Sabbatini G., Pezzi A. (2024). Presepsin in Critical Illness: Current Knowledge and Future Perspectives. Diagnostics.

[28] Ghenu M.I., Dragoş D., Manea M.M., Ionescu D., Negreanu L. (2022). Pathophysiology of sepsis-induced cholestasis: A review. JGH Open.

[29] Xu J., Legido-Quigley C., Ma Y. (2023). Metabolomics Applied to Biomarker Discovery in Liver Related Diseases. Ph.D. Thesis.

[30] Ghoneim E.M., ElAziz A.M.A., AbuAmer A.M., Ghonaim A.M., Awad S.M. (2024). Value of Soluble CD14 (Presepsin) in Diagnosis of Bacterial Sepsis in Patients with Chronic Liver Disease. Egypt. J. Med. Microbiol..

[31] Zhang Z., Ma K., Yang Z., Cheng Q., Hu X., Liu M., Liu Y., Liu T., Zhang M., Luo X. (2021). Development and Validation of a Clinical Predictive Model for Bacterial Infection in Hepatitis B Virus-Related Acute-on-Chronic Liver Failure. Infect. Dis. Ther..

[32] Jang J.H., Choi E., Kim T., Yeo H.J., Jeon D., Kim Y.S., Cho W.H. (2024). Navigating the Modern Landscape of Sepsis: Advances in Diagnosis and Treatment. Int. J. Mol. Sci..

[33] Matuszak S.S., Kolodziej L., Micek S., Kollef M. (2025). Antibiotic De-Escalation in the Intensive Care Unit: Rationale and Potential Strategies. Antibiotics.

